# Seizure-Induced Resolution of Depressive and Psychotic Symptoms in a Pregnant Patient: A Case of Spontaneous Neuromodulation

**DOI:** 10.7759/cureus.110418

**Published:** 2026-06-07

**Authors:** Ariel E Sosa Gomez, Ryan M Go, David Valdez

**Affiliations:** 1 Psychiatry, University of Texas Rio Grande Valley, Harlingen, USA; 2 Faculty of Medicine and Surgery, University of Santo Tomas, Manila, PHL

**Keywords:** generalized tonic clonic seizures, major depressive disorder (mdd), neuromodulation therapy, peripartum psychosis, suicide ideation

## Abstract

Many individuals with epilepsy often develop major depressive disorder (MDD), creating a complex interplay that exacerbates outcomes for affected individuals. The relationship between these disorders may be mediated by overlapping neurobiological mechanisms, including disturbances in the limbic system, neurotransmitter imbalances, and the psychosocial stressors of chronic illness. While the co-occurrence typically leads to a worsening of symptoms, rare instances have been reported in which seizure activity appears to ameliorate psychiatric symptomatology. We present the case of a 23-year-old pregnant Hispanic female with a longstanding history of treatment-resistant depression with psychotic features and epilepsy, who demonstrated full remission of depressive and psychotic symptoms following a generalized tonic-clonic seizure. The patient, previously admitted to a psychiatric state center due to severe depressive symptoms and self-injurious behaviors, was transferred to our institution following seizure onset for medical stabilization. Remarkably, psychiatric evaluation revealed no evidence of current psychopathology. This case emphasizes the potential neuromodulatory effects of ictal activity and draws parallels to the therapeutic mechanisms of electroconvulsive therapy (ECT), raising important considerations for the intersection of neurology and psychiatry. Further investigation into the antidepressant effects of seizure activity may offer insights into novel treatment avenues for refractory psychiatric disorders.

## Introduction

Epilepsy and major depressive disorder are two ailments that are usually found together with a reciprocal relationship that worsens the outcomes in affected individuals. Various research and articles have demonstrated that patients with epilepsy are prone to develop depressive symptoms compared to the general population [1]. It was reported in a meta-analysis done in 2022 that the merged prevalence of depression in patients who have epilepsy was 27% in population-based settings and 34% in clinical settings, emphasizing the elevated burden this comorbidity may inflict on patient health [2]. The relationship between these two comorbidities may be influenced by shared neurobiological pathways and mechanisms that include disturbance in limbic circuits, neurotransmitter unevenness, and the psychosocial impact of chronic illness [3,4].

The relationship between psychiatric symptoms and seizure activity is complex and interdependent, as each influences the other. However, it is also known that seizures may positively alter the trajectory of mood disorders and other psychopathologies [5]. There have been some instances and rare occasions in which depressive symptoms subsided after non-induced seizure activity, raising questions about the neuromodulation effects of ictal activity on affective states [6]. 

We describe the case of a pregnant patient with an extensive history of epilepsy and depressive disorder with psychotic features, who, after undergoing a generalized tonic-clonic seizure, experienced remission of her severe depressive symptoms and psychosis. With this case, we intend to highlight the potential interplay between mood regulation and seizure activity and contribute to the literature studying the association between neurobiological and psychiatric conditions.

## Case presentation

We present the case of a 23-year-old pregnant Hispanic woman at 32 weeks’ gestation with a complex psychiatric and neurological history, including a seizure disorder, depressive disorder with psychosis, multiple prior suicide attempts, and recurrent psychiatric hospitalizations.

The patient’s psychiatric history is notable for two serious suicide attempts - one in 2017 involving multiple episodes of wrist-cutting and overdose, and another in 2019, when she attempted to throw herself from a moving vehicle. She has been admitted to inpatient psychiatric facilities on multiple occasions; her most recent hospitalization at a state psychiatric center, immediately before admission to our hospital, was due to self-injurious behavior, including choking herself and punching her abdomen. These behaviors reportedly occurred in the context of severe depression and psychosis, including auditory hallucinations, particularly during a period of incarceration. 

Within the first 48 hours of admission at the psychiatric facility, the patient had an acute seizure episode and was subsequently transferred to our tertiary care hospital for further medical evaluation and stabilization. Upon arrival, she received intravenous levetiracetam (Keppra) 500 mg for acute seizure management and was temporarily transferred to the intensive care unit due to concerns regarding hemodynamic instability, including diastolic readings dropping into the 50s. 

Laboratory and imaging workup included a complete metabolic panel (CMP), complete blood count (CBC), urine drug screen (UDS), and a non-contrast magnetic resonance imaging (MRI) scan of the brain - all of which did not appear to contribute to the patient's seizure activity or mental health symptomatology (Tables 1-2).

**Table 1:** Urine drug screen on admission. *Results are from urine drug screening performed within 24 hours of hospital admission, except for the levetiracetam level, which was measured on the fourth day of hospitalization (August 25, 2024). NV, normal value

Urine drug screen	Result
Amphetamine	Negative
Barbiturates	Negative
Benzodiazepine	Negative
Cocaine	Negative
Ethanol	Negative
Methadone	Negative
Opiates	Negative
Oxycodone	Negative
Phencyclidine	Negative
Tetrahydrocannabinol	Negative
Levetiracetam (NV = 10-40 mcg/mL)	65.4*

**Table 2:** Patient laboratory results. NV, normal value; CO_2_, carbon dioxide; BUN, blood urea nitrogen; EGFR, estimated glomerular filtration rate; ALP, alkaline phosphatase; ALT, alanine aminotransferase; AST, aspartate aminotransferase

Laboratories	August 8, 2024	August 24, 2024	August 25, 2024
Glucose (NV = 70-99 mg/dL)	93	63	64
Sodium (NV = 135-145 mmol/L)	137	140	140
Potassium (NV = 3.6-5.2 mmol/L)	3.2	3.1	3.6
Chloride (NV = 96-106 mEq/L)	110	112	109
CO2 (NV = 20-30 mmol/L)	21	22	27
Calcium (NV = 8.5-10.2 mg/dL)	7.8	7.8	7.9
BUN (NV = 6-21 mg/dL)	8	4	5
Creatinine (NV = 0.5-1.1 mg/dL)	0.48	0.38	0.36
EGFR (NV = >90 mL/minute/1.73 m²)	>90	>90	>90
Albumin (NV = 3.5-5.5 g/dL)	2.8	2.8	2.6
ALP (NV = 35-104 IU/L)	69	67	71
ALT (NV = 4-36 U/L)	11	11	11
AST (NV = 8-33 U/L)	16	18	16
Bilirubin (NV = 0.1-1.2 mg/dL)	0.4	0.5	0.3
Total protein (NV = 6-8.3 g/dL)	4.9	5.0	4.7

A 24-hour electroencephalogram (EEG) revealed mild abnormalities, specifically background slowing, but no interictal epileptiform discharges. 

At the time of psychiatric evaluation, the patient was alert, oriented, and hemodynamically stable. She was observed resting calmly in her hospital bed and engaged appropriately with the evaluating team. She was cooperative, pleasant, and exhibited no overt signs of internal preoccupation or active psychosis. She was dressed in hospital attire and was oriented to person, time, place, and situation. Her speech was spontaneous, with normal rate and prosody, and her thought processes were logical, organized, and goal-directed. Her affect was reactive and congruent with her described euthymic mood. Cognition, attention, and judgment appeared intact. 

The patient acknowledged experiencing seizures while in the psychiatric facility but denied awareness of the behaviors that led to her admission there. She denied recent symptoms of depression, suicidal ideation, auditory hallucinations, or other psychotic features. Notably, she acknowledged experiencing depression during her adolescence, which she attributed to a history of sexual abuse by her father and physical abuse by her mother. However, she was unable to provide further details regarding past psychiatric hospitalizations or suicide attempts. 

Psychometric screening using the Patient Health Questionnaire-9 (PHQ-9) [7] yielded a score of 0, indicating no current depressive symptoms. The Brief Psychiatric Rating Scale (BPRS) [8] score was 18, further supporting the absence of acute psychiatric pathology at the time of evaluation. 

Medication reconciliation revealed that, before her seizure and transfer, the patient was prescribed fluoxetine 40 mg daily, olanzapine 15 mg daily, and levetiracetam 1,000 mg twice daily. However, records suggested poor adherence to her psychotropic regimen, both in the psychiatric facility and while incarcerated. This likely contributed to a deterioration in both her seizure control and psychiatric stability. At the time of evaluation at our hospital, she was receiving levetiracetam 1,500 mg intravenously twice daily, along with supportive medications and PRN medications as needed. No antipsychotic or antidepressant therapy was being administered at that time. 

Given the patient’s clinical improvement, absence of active psychiatric symptoms, and current pregnancy, the treatment team recommended withholding psychotropic medications during hospitalization to minimize fetal exposure. It was suggested that re-initiation of psychiatric medications - potentially beginning with a low-dose selective serotonin reuptake inhibitor (e.g., sertraline) - be considered after delivery or once the patient returned to a more restrictive and structured environment, such as the psychiatric state hospital. In summary, since admission to our tertiary hospital, the patient remained psychiatrically symptom-free throughout her five-day hospital stay. She was subsequently discharged back to the state psychiatric hospital for further monitoring and longitudinal care.

## Discussion

This case presents a remarkable outcome following a generalized tonic-clonic seizure in a patient with a long-standing history of severe depression, psychosis, and multiple suicide attempts. Notably, the patient experienced a resolution of psychiatric symptoms post-seizure. Similar cases have been reported in the literature, where seizure activity has led to transient or sustained improvements in psychiatric conditions, suggesting a potential neuromodulatory effect of seizures [9,10]. 

Levetiracetam (Keppra), a commonly used antiepileptic drug (AED), was effective in managing the patient’s seizure activity upon hospital admission [11]. A key consideration in this case was the possibility of functional seizure-like episodes occurring alongside her known epileptic disorder. While such a presentation is uncommon, it is not implausible in individuals with comorbid psychiatric conditions, particularly those susceptible to Functional Neurological Symptom Disorder (FNSD) [12]. However, the patient’s clinical history, multiple EEGs over the past decade, and the consistent presentation of tonic-clonic seizures strongly supported the diagnosis of a true seizure disorder over FNSD. 

Although valproic acid could have been considered as an alternative AED due to its mood-stabilizing properties [13], its known teratogenicity made it an inappropriate choice in this pregnant patient [14]. 

The sudden resolution of psychiatric symptoms post-seizure bears similarity to the effects observed with electroconvulsive therapy (ECT), a well-established treatment for severe depression. ECT functions by inducing controlled seizures, which are believed to trigger neuroplastic changes, normalize dysregulated neurotransmitter systems, and restore connectivity in mood-related neural circuits, particularly within the prefrontal-limbic network [15]. It is plausible that a similar neurobiological mechanism underlies the symptom resolution seen in this patient.

Given the patient’s stable mental status and lack of depressive or psychotic symptoms at the time of evaluation, no psychotropic medications were initiated. Although long-term use of antidepressants is known to be critical for relapse prevention in patients with recurrent depression [16], the decision was made to avoid initiating such agents during pregnancy due to potential fetal risks. These include persistent pulmonary hypertension of the newborn (PPHN), neonatal adaptation syndrome, and miscarriage [17-19], among others. Additionally, while the seizure risk associated with antidepressants is generally minimal, certain agents have been shown to lower the seizure threshold [20-22]. 

Initiation of antipsychotic medication was similarly deemed unnecessary, as the patient was not exhibiting any active psychotic symptoms. The patient remained psychiatrically stable for five days post-admission and was subsequently transferred back to the psychiatric facility with recommendations to follow up with her psychiatrist and consider re-evaluation for antidepressant therapy postpartum or upon clinical indication.

## Conclusions

This case underscores the rare but intriguing phenomenon of spontaneous resolution of psychiatric symptoms following seizure activity in a patient with treatment-resistant depression and psychosis. The patient's improvement parallels mechanisms observed in electroconvulsive therapy, suggesting that generalized seizures may trigger neurobiological changes capable of stabilizing mood and alleviating psychopathology. While antiepileptic treatment with levetiracetam successfully controlled seizures, the decision to withhold psychotropics was guided by the patient's current mental status, pregnancy, and the desire to minimize fetal exposure. This case contributes to the growing body of literature exploring the bidirectional relationship between epilepsy and psychiatric illness and supports the hypothesis that seizure activity may hold therapeutic value in select cases. However, we acknowledge the absence of baseline psychometric measurements prior to the seizure, limited follow-up (five days), and potential confounding factors such as environmental change, sleep restoration, or postictal effects. It is important also to note that this is a single-case design with a short observation window indicating the need for further studies. Ongoing research into the neurobiological underpinnings of this relationship may further elucidate novel strategies for managing comorbid depression in patients with epilepsy.
